# Conflict between cattle ranching and the conservation of jaguar (Panthera onca) and puma (Puma concolor) in the Amazon arc of deforestation

**DOI:** 10.1371/journal.pone.0312077

**Published:** 2024-11-20

**Authors:** Vanessa Díaz-Vaquero, Nuno Negrões, Carlos Fonseca, Leandro Silveira, Anah Tereza Jácomo, Mario Quevedo, Eloy Revilla

**Affiliations:** 1 Department of Biology of Organisms and Systems, University of Oviedo, Oviedo, Spain; 2 Biodiversity Research Institute (IMIB, CSIC-UO-PA), University of Oviedo, Campus Mieres, Mieres, Spain; 3 Department of Conservation Biology, Estación Biológica de Doñana CSIC, Seville, Spain; 4 CESAM & Biology Department- Aveiro University, Campus Universitário de Santiago, Aveiro, Portugal; 5 Jaguar Conservation Fund/Instituto Onça-Pintada, Mineiros, Goáis, Brazil; Universidade Federal de Mato Grosso do Sul, BRAZIL

## Abstract

Livestock predation constitutes the primary source of conflict between humans and large carnivores. Moreover, human factors, such as attitudes and emotions, can affect people’s tolerance towards carnivores, exacerbating the conflict. Such conflicts often lead to retaliatory killing of carnivores, which not only poses significant threats to species conservation but also to ecosystem functioning and services. Therefore, mitigating human-carnivore conflicts is essential to promote both species conservation and human well-being. Here, we studied the conflict between extensive cattle ranching and the conservation of jaguars (*Panthera onca*) and pumas (*Puma concolor*) in 129 ranches located in the Amazon arc of deforestation. We interviewed ranchers about livestock management procedures, livestock mortality, approaches to reduce predation in the area, and attitudes regarding carnivores and conservation. Our results revealed that ranchers did not perceive carnivore attacks as the primary cause of cattle mortality. However, they exhibited a significant lack of tolerance towards these incidents, partially explained by economic reasons. They also showed negative attitudes towards big cats, which were mainly associated with social factors, such as low educational background. As a consequence, jaguar and puma were frequently killed in retaliation. Ranches showed different vulnerability to attacks depending on cattle management (cattle density, calves) and landscape (forest cover inside the ranch, distance to national parks). Our findings suggest that the conflict between cattle ranching and big cats conservation in the Amazon deforestation frontier is trigger by livestock predation, but perpetuated by limited knowledge about carnivores, the lack of support from the government to mitigate livestock losses, and the perception that conservation laws conditioned the viability of cattle ranching. Forthcoming mitigation strategies should focus on interventions designed to increase people’s tolerance towards jaguars and pumas (e.g. improving knowledge about ecosystem services provided by large carnivores).

## Introduction

Humans have transformed primary ecosystems into highly modified agricultural and urban areas [1]. This encroachment of people in natural habitats, driven by population growth and increasing demand for natural resources [2], has led to a growing number of human-wildlife interactions. Negative interactions between people and wildlife pose a serious threat to nature conservation [3] as the species involved are often persecuted and killed by people. This results in the contraction of their ranges or even the extirpation of their populations [4]. Consequently, negative interactions between people and wildlife emerge as major conservation challenges, which that need to be mitigated to ensure long-term species conservation together with the welfare of human societies that share space with them.

Predation of livestock is a major source of conflict between people and large carnivores [5]. A wide variety of prevention measures, such us electric fences or guarding dogs, exist to deal with carnivore attacks on livestock. However, their effectiveness varies greatly depending on the species and context [6, 7]. Furthermore, conflicts with large carnivores may remain even after implementing mitigation measures aimed at increasing tolerance towards species, such as economic compensation [8] or culling [9]. This persistence of the problem despite the application of preventive and mitigation measures points out the importance of human aspects in fuelling conflict [10].

Retaliatory killing of carnivores is a frequently response to livestock predation. Species killing may be responsible for up to 85% of deaths in some populations of large carnivores [11], potentially leading to extirpation of populations [12], or even the species extinction [13]. Such outcomes will have important implications for ecosystem functioning [14, 15], as well as for the ecosystem services that large carnivores provide to humans [16]. Human-carnivore conflicts are heterogeneous in nature, making it difficult to generalise solutions across study cases [17]. In addition, poorly fitted management strategies for a particular case may ultimately lead to an exacerbation of the problem [18]. A multidisciplinary approach that include both ecological and social perspectives of the problem is therefore needed to obtain a comprehensive understanding of human-carnivore conflicts [19], and to facilitate coexistence among people and large carnivores [20].

We studied the ongoing conflict between extensive cattle ranching and the conservation of jaguars (*Panthera onca*) and pumas (*Puma concolor*) in the Brazilian Amazon deforestation frontier. In this biodiversity hotspot [21], livestock predation by jaguars and pumas is frequent [22], and preventive measures such as electric fences, or the inclusion of creole cattle and water buffalos has proved to be effective against livestock predation by carnivores, especially in small ranches [23]. However, habitat degradation caused by cattle ranching expansion [24] together with retaliatory killing still remains important factors of large carnivores decline in the area, especially for the jaguar [25, 26]. Recent studies have shown that the current jaguar range is 14% less than the estimate for 2015 [27]. One of the causes of this rapid decline is the expansion of cattle ranching, which is often associated with deforestation and retaliatory killings, the combined effects of which can lead to jaguar extirpation [28, 29]. Conflict mitigation in this region is therefore essential to promote the long-term conservation of jaguars and pumas. The aim of this study was to obtain a comprehensive understanding to the conflict, by looking at the causes of livestock mortality, the factors influencing livestock predation, and the attitudes of livestock owners. Our hypotheses were that (I) livestock predation is not the main cause of cattle mortality, (II) both landscape attributes and livestock management practices are important in livestock predation by large carnivores, and (III) ranchers’ attitudes towards large carnivores are influenced not only by previous predation incidents, but also by social factors.

## Material and methods

### Study area

**Fig 1:**
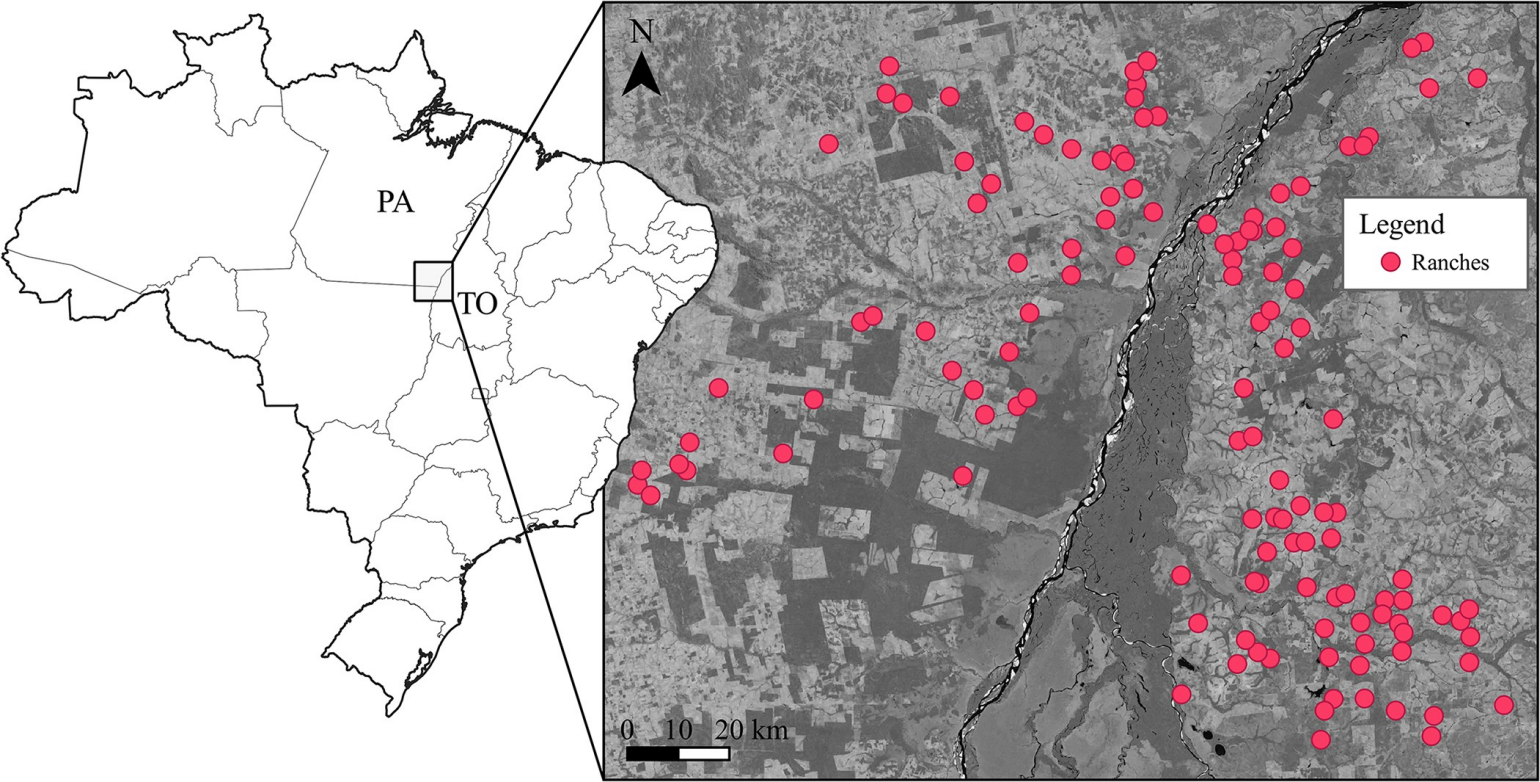
Study area map. Red circles indicate ranches surveyed in 2008 in the Brazilian states of Pará (PA) and Tocantins (TO). Satellite image of the study area was obtained from Landsat 7, with forest areas shown in dark grey. Country and state boundaries were obtained from Natural Earth Data.

The study was carried out in a partially deforested region of approximately 19,000 km^2^ in the transition zone between the Cerrado and the Amazon biomes (Fig 1). The landscape in the study area was dominated by moist broadleaf forests in the Amazon biome, and tropical and subtropical grasslands in the Cerrado biome. The primary economic activities were cattle ranching and agriculture [30]. Amazon and Cerrado are biomes that harbour high biological diversity [21] and may provide essential ecosystem services, such as disease mitigation and local climate regulation [31]. These regions have been vastly affected by deforestation [32] due to agricultural expansion [33, 34]. Cattle ranching expansion [24] was favoured by the construction of Trans-Amazon highway [35] that facilitated access to intact forests, and by the government policies that fostered land tenure and speculation [36]. This, together with the eradication of livestock diseases [37] and technological developments in beef production [38] positioned Brazil as the world’s largest beef exporter [39]. To guarantee preservation of tropical forests, Brazilian legislation (No. 4,771, of September 15, 1965) made it mandatory to preserve a proportion of woodland, known as legal reserves, on privately owned land. In 2001 (No. 2,166–67, of August 24, 2001), an interim measure was implemented, requiring the preservation of 80% of woodland in the Legal Amazon, and 20% of woodland in the Cerrado biome.

### Species

The jaguar has been classified as Near Threatened by the IUCN [40] and many subpopulations are endangered or critically endangered. The Amazon basin, covering 79% of the species’ global range, is its largest remaining stronghold, and as such is critical for jaguar conservation [41]. The puma has been classified globally as Least Concern by the IUCN [42], and regionally Vulnerable by the Red List of Endangered Species of Brazil [43]. However, puma density in the Amazon basin and the degree of connectivity between local populations remains poorly known [44].

### Data collection

We visited 129 ranches and interviewed ranch managers, from 01/01/2008 to 31/12/2008, using a structured questionnaire (S1 and S2 Files) with yes/no, open, one-choice, and multiple-choice questions [45]. We asked to each ranch manager about ranch characteristics, cattle management techniques, cattle mortality, approaches to reduce predation in the area, and attitudes regarding carnivores and conservation. We obtained information about the respondent, including their age, education level, state of origin, length of time living in the region, and length of time working on the ranch (S3 File). Ethics committee approval was not obtained prior to the start of the study as it was not mandatory in Brazil at the time of designing the study (National Health Council Resolution No. 196/96, of October 10, 1996). We conducted all interviews following a Free, Prior, and Informed Consent framework. Before the beginning of each questionnaire, participants were presented with an informed consent form, and they were asked by the interviewer to also express their consent orally (due to low literacy). If respondents were under the legal age of 18, we obtained parental or guardian consent. Participation was voluntary and anonymous. Participants could withdraw from questionnaires at any time during the survey, and their responses were deleted.

### Data analysis

To assess the reliability of survey data on the causes of cattle mortality, we compared the mortality perceived by the surveyed ranchers with the detailed mortality recorded in the logbooks for two ranches (S4 File). To identify potential problems with biased responses we compared the data collected on the presence of predated cattle on ranches (0/1) and the number of predated heads with the respondent profile (i.e. age, education level, length of time living in the region, and length of time working on the ranch).

To identify which factors influenced livestock predation by jaguar and puma, we fitted two different generalized linear models (GLM): a binomial GLM on the occurrence of cattle predation on ranches to evaluate disparities in ranch vulnerability, and a beta-binomial GLM on the proportion of cattle predated on those ranches that had predations to identify which factors were associated with a higher impact of carnivores on the herd. In the latter model, herd size was included as prior weights. We included as predictors a total of six landscape variables and seven cattle management variables (see Table 1 for variables description). Due to the high number of predictors included (13) and the limited number of ranches visited, it was not advisable fitting a single model with all predictors (landscape + management). Consequently, we fitted on each response variable (occurrence of predations, proportion of cattle predated) two different models: one based on landscape variables, and another based on cattle management variables (i.e. four models in total). The number of ranches used to fit the four models ranged from 80 to 119, depending on the predictors included (see S5 File for details). Prior to analysis, quantitative predictor variables with skewed distributions were log-transformed and all quantitative predictor variables were scaled (mean = 0, sd = 1). We did not find multicollinearity among predictors according to the variance inflation factor (VIF < 3) [46]. No residual spatial autocorrelation was detected in any of the fitted models. For each of the four models fitted, we selected candidate models with ΔAIC_c_ < 2 (S5 File) and computed the average model. We did not include the variable *Breeding* (0/1) as predictor when modelling the proportion of cattle predated in attacked ranches due to the majority of them (97%) had included this phase of production on their operations.

**Table 1:** Description of landscape and cattle management variables used to fit models on cattle predation by jaguar and puma.

**Landscape variables**	
Forest	Percentage of forest cover from the total area of the ranch. It represents carnivore’s habitat.
Forest3	Average percentage of forest cover in the three nearest ranches. This measure was used as a proxy of surrounding landscape to account for spatial autocorrelation between observations.
River	Euclidean distance (km) between the ranch and the nearest water course. It represents carnivore’s habitat, especially for jaguars which prefer riparian forests, and water sources for wild and domestic animals.
National Park	Euclidean distance (km) between the ranch and the boundary of the nearest national park (category II in IUCN Protected Areas). Ecological functions and native species composition are relatively intact in these areas, acting as a refuge for jaguars and pumas.
Road	Euclidean distance (km) between the ranch and the nearest road. Roads could be used by carnivores to move through their territory, but also could indicate areas where human-induced mortality would be greater.
City	Euclidean distance (km) between the ranch and the nearest city or village. It could represent areas where human-induced mortality would be greater.
**Management variables**	
Density	Number of cattle heads by km^2^ of pasture.
Workers	Number of workers per 100 heads of cattle. Workers were a proxy of the intensity of cattle management in the ranch.
Subdivisions	Number of subdivisions per 100 heads of cattle. Subdivisions are ranch areas with fences, where cattle stay during some periods of the year. The main objective of subdivisions is to control grazing intensity. They can include inside a ranchers’ house.
Breeding	Categorical variable with two levels (0/1). This type of management involves pregnant cows and calves in the herd. It represents the presence of vulnerable individuals (calves) to predation.
Growing	Categorical variable with two levels (0/1). This type of cattle management implies yearlings in the herd, which are supplementary fed with nutrients that the pasture is deficient in, often during dry season. Yearlings are brought to adequate pastures in each growing stage or workers provided them fodder to growing fast. It represents the presence in the herd of individuals (yearlings) less vulnerable to predation.
Fattening	Categorical variable with two levels (0/1). This type of cattle management implies adults in the herd which are artificially fed to reach their maximum weight for meat production. Adults are less vulnerable to predation, and they can have some defensive behaviour against carnivores.
Maternity	Categorical variable with two levels (0/1). It represented fenced areas in the ranch where calves and mothers stayed inside to limit calves’ movements and ensure their survival. This variable could represent a type of management for preventing livestock predation.

To identify if ranchers’ attitudes were associated to the impact of large carnivores on their ranches, we compared their attitudes towards livestock predation and carnivore conservation with the presence of cattle predated on ranches (0/1) and the proportion of the herd affected in attacked ranches. In addition, we compared the number of predations that respondents were willing to tolerate with the size of their herd, the presence of cattle predated on the ranch (0/1), and the proportion of animals predated in those ranches that experienced cattle predation by carnivores. We did not include in this comparison ranches with less than 50 animals in the herd (n = 6), due to response options ranged from less than 5 individuals to more than 50 individuals. We finally tested whether there was any correlation between the attitudes showed by ranch mangers and their age, education level, length of time living in the region, and length of time working on the ranch. We used Kruskal-Wallis, Mann-Whitney U, Chi-square and Fisher tests for the analyses. We used R software version 4.1.0 [47].

## Results

Ranch size ranged from 24 to 103,000 ha (median = 2,807 ha), with forest cover varying from 0 to 96% (median = 41%). Herd size within ranches ranged from 2 to 17,000 heads (median = 1,058) and the median cattle density in pastures was 0.86 heads / ha. Number of workers per ranch ranged from 1 to 75 (median = 3). All of the ranches engaged in extensive grazing, with 92% including a breeding phase, 67% a growing phase, and 53% a fattening phase in their operation (refer to Table 1 for definitions). Additionally, 66% of the ranches had a maternity paddock for calving cows and their calves.

**Fig 2:**
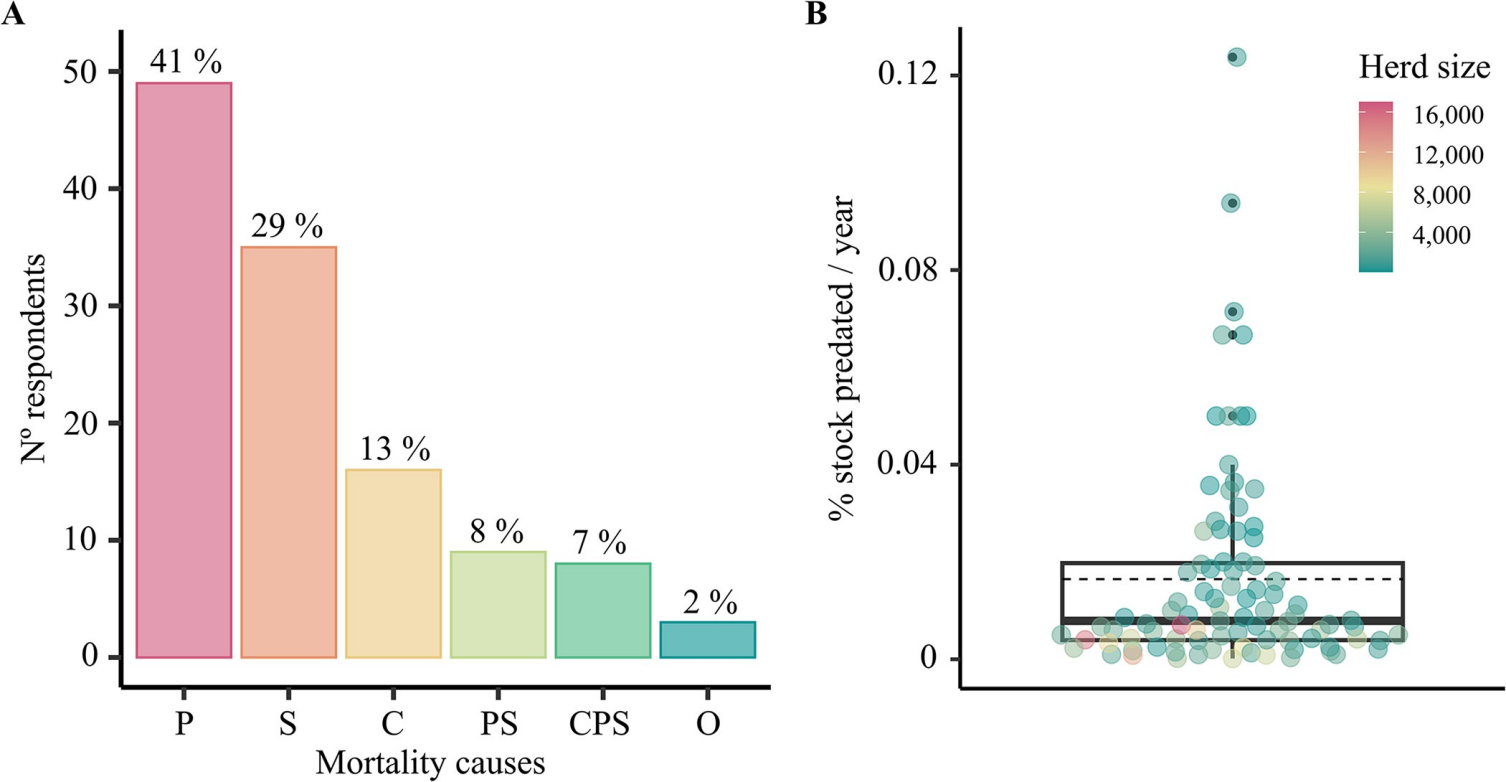
Impact of jaguar and puma on cattle ranching. (A) Sources of cattle mortality perceived by ranch managers as the most significant in their ranches. P: ingestion of toxic plant, S: snake bite, C: predation by jaguar and puma, PS: ingestion of toxic plant and snake bite, CPS: predation by jaguar and puma, ingestion of toxic plant and snake bite, O: other causes. The percentage of respondents appears on the top of each bar. (B) Percentage of cattle stock annually predated by jaguar and puma in ranches with cattle predation problems (n = 91). Values were calculated as the perceived number of animals annually predated divided by the herd size reported by ranch managers. The box plot shows the data distribution with outliers (black dots) and mean (dashed line). Raw data values coloured by herd size was added to the plot.

When asked about cattle mortality, 41% of ranch managers identified the ingestion of toxic plants as the primary cause of death on their ranches (Fig 2A). Jaguars were reported to be present on 75% of ranches (46% by direct sightings, and 54% by signs), and cattle predation by jaguar and puma was reported on 71% of the ranches. Ranch managers who had worked on a ranch for longer periods reported cattle predation problems more frequently (χ^2^ test = 7.38, df = 2, P = 0.028). On ranches where cattle were predated by jaguar and puma, 28% of ranch managers perceived predation as the main cause of cattle mortality. The average number of heads predated per year and per ranch varied from 1 to 120, which translates to 0.01% - 12.4% of the herd size being affected (Fig 2B). The perceived values obtained from the ranchers’ memory about cattle mortality were consistent with those values obtained from the detailed data in the logbooks (S4 File) and were not influenced by the respondents’ profile. Most ranchers (99%) reported that jaguars and pumas frequently attacked young cattle, and blamed jaguars instead of pumas for most predations (86 and 14%, respectively).

The occurrence of cattle predation on ranches was related to cattle management: ranches including a breeding phase, and with higher cattle density, were more likely to report cattle predation by big cats (Table 2). We did not find a relationship between the landscape variables and the presence of cattle predated by jaguar and puma on ranches. The impact of jaguar and puma on the herd was associated with the landscape: ranches with greater proportion of forest cover and close to national parks showed greater proportion of herd predated (Table 2). We did not find a relationship between cattle management variables and the proportion of herd predated.

**Table 2:** Model results about cattle predation by jaguars and pumas on ranches.

*Presence (0/1) of cattle predated by jaguar and puma on ranches*					
Model	Variables	Estimate (95% CI)^a^	Std. Error^b^	*P* ^c^	*w*_*i*_ ^d^
Landscape	*City*	0.414 (-0.010, 0.839)	0.214	0.056	1
n = 119	*River*	-0.183 (-0.623, 0.258)	0.224	0.417	0.56
	*Forest*	0.107 (-0.261, 0.475)	0.187	0.570	0.42
Management	***Breeding***	**2.629 (0.702, 4.556)**	**0.973**	**0.007**	**1**
n = 115	***Density***	**0.727 (0.129, 1.326)**	**0.302**	**0.017**	**1**
	*Subdivisions*	1.061 (-0.124, 2.245)	0.568	0.079	1
	*Fattening*	0.479 (-0.634, 1.593)	0.565	0.399	0.59
	*Maternity*	0.363 (-0.700, 1.425)	0.539	0.504	0.47
	*Workers*	-0.116 (-0.623, 0.391)	0.257	0.654	0.33
	*Growing*	-0.056 (-0.559, 0.447)	0.255	0.826	0.13
*Proportion of cattle predated in attacked ranches*					
Model	Variables	Estimate (95% CI)^a^	Std. Error^b^	*P* ^c^	*w*_*i*_^d^
Landscape	***Forest***	**0.273 (0.051, 0.495)**	**0.112**	**0.016**	**1**
n = 83	***National Park***	**-0.231 (-0.443, -0.020)**	0.106	**0.032**	**1**
	*Forest3*	0.070 (-0.138, 0.278)	0.105	0.508	0.46
	*River*	-0.040 (-0.200, 0.119)	0.081	0.621	0.37
Management	*Workers*	0.160 (-0.094, 0.414)	0.128	0.216	0.77
n = 80	*Fattening*	-0.298 (-0.826, 0.230)	0.268	0.269	0.70
	*Growing*	-0.220 (-0.741, 0.300)	0.264	0.407	0.55
	*Density*	-0.094 (-0.321, 0.134)	0.115	0.421	0.54
	*Maternity*	-0.090 (-0.459, 0.279)	0.187	0.634	0.30
	*Subdivisions*	0.020 (-0.095, 0.135)	0.058	0.732	0.18

Model selection was performed separately for landscape and management variables. Candidate models with ΔAIC < 2 were retained and they were summarized in an average model (see Material and methods and S5 File for further details). Variables with p-value < 0.05 appear in bold font. n: sample size.

^a^95% CI, 95% confident interval of the estimate

^b^Std. Error, standard error of the estimate value

^c^*P*, p-value for the z-score

^d^*w*_*i*_, Akaike weight for each variable

Asked about the approaches used to reduce predation in the area, ranchers said that changes in cattle management were prevalent (76%), followed by shooting (50%), poisoning (8%), others (8%), and cage traps (1%). Ranchers perceived economic compensation (50%), followed by translocation and culling (35% and 13%, respectively), as the best management options to reduce cattle predation. Ranchers that deal with cattle predated by jaguar and puma on their ranches preferred cattle management (χ^2^ = 9.12, df = 1, *P* = 0.003) and the payment of damages (χ^2^ = 4.27, df = 1, *P* = 0.039) as the best responses to cattle predation.

Ranchers showed contradictory attitudes regarding nature conservation and livestock predation (Table 3). For instance, the majority of respondents considered nature and wildlife as a treasure that should be preserved, but 52% of them considered that laws preserving nature were holding back the region’s development. The protection of jaguars was perceived as necessary by 85% of respondents, and yet 42% would be happier if jaguars disappeared from the area. Jaguars and pumas were perceived as a threat to cattle (70%), and to a lesser extent also as a threat to people (37%). Indeed, 12% of respondents reported they were aware of attacks on humans. Most ranchers perceived levels of cattle predation as an inherent, acceptable risk in cattle business (88%), but half of them did not tolerate attacks in their ranches, partly because some ran purebred cattle. In fact, 68% of ranch managers would not accept to lose more than five head of cattle per year. Ranchers with larger herd sizes tolerated a greater number of losses (Kruskal Wallis, H = 9.22, df = 2, *P* = 0.010). Ranchers considered that their ranches are doing well (84%). However, they would like to receive help to solve cattle predation on their ranches (90%), and considered that cattle predation by jaguar and pumas should be addressed by authorities (83%) rather than individual ranches (24%).

**Table 3:** Ranchers’ attitudes towards big cats and conservation. The number of respondents (n) and the percentage obtained for each response are shown.

Attitude statement	n	Disagree (%)	Do not know (%)	Agree (%)
Jaguars are a threat to cattle	128	29	2	70
Jaguars are a threat to people	128	62	1	37
We cannot tolerate livestock predation by jaguars at this ranch	128	48	2	50
Livestock predation is an acceptable and natural risk in cattle business	128	12	1	88
I would be happier if there were no more jaguars around	128	54	4	42
Jaguars need to be protected	128	13	2	85
It is necessary to find a solution for livestock predation	128	9	1	90
Things are going well in this ranch	128	16	-	84
Livestock predation should be addressed by authorities	128	12	5	83
Livestock predation should be addressed by each ranch, without external help	128	75	1	24
I would like to receive help to solve the problem of predation at this ranch	128	4	2	95
Nature/Wildlife of the region is a treasure to be preserved by all people	128	-	1	99
I am concerned about nature/wildlife conservation in the region	128	7	2	91
Laws that preserve nature hinder the development of the region	128	44	5	52

**Fig 3:**
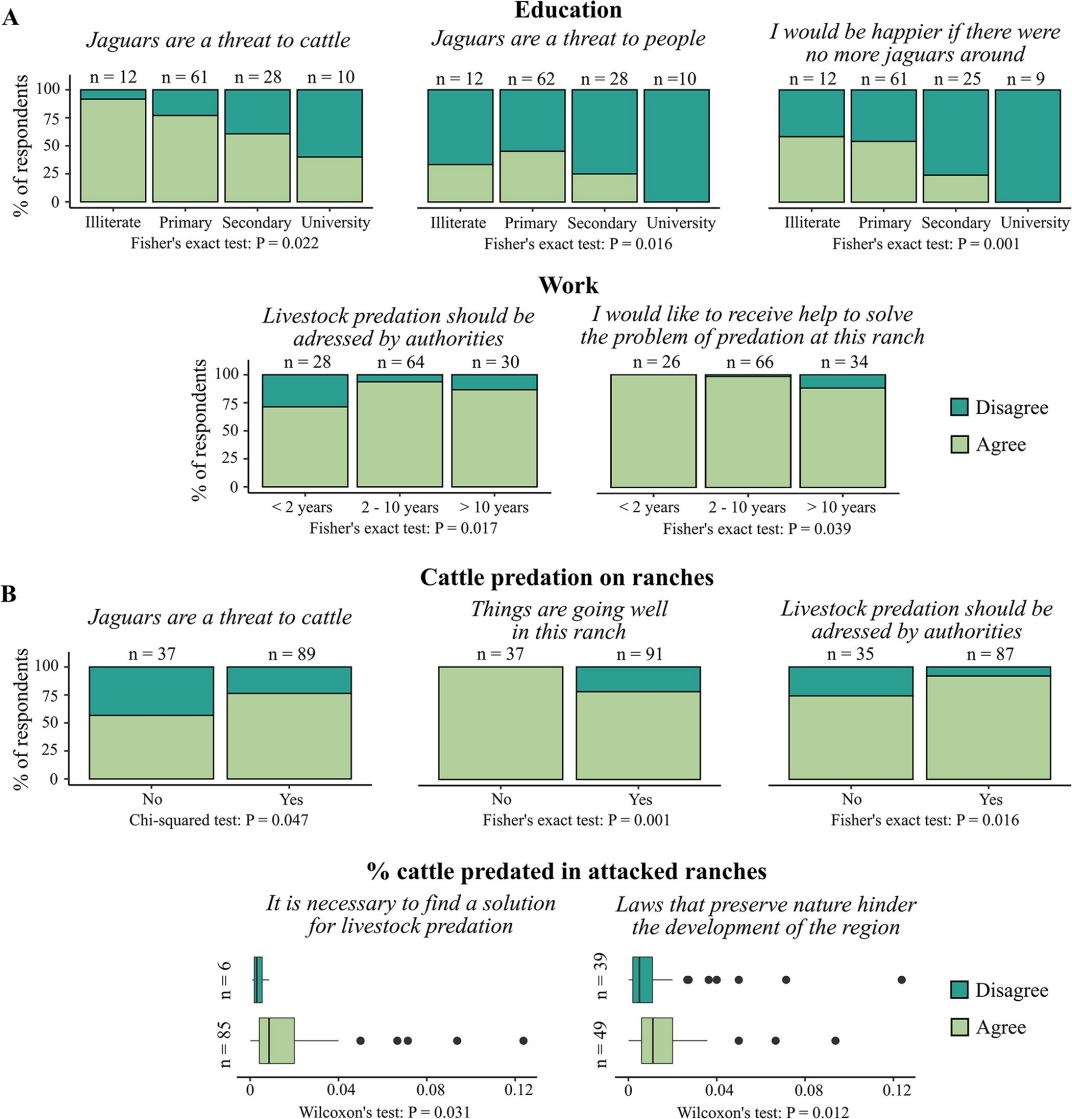
Factors influencing ranchers’ attitudes. (A) Rancher profile. (B) Predation on the ranch. Each graph shows the attitude statement, the total number of respondents at each level (n), and the p-value for the corresponding statistical test (P). “Do not know” option was removed from the graphs (and the subsequent analysis) for clarity.

Attitudes varied depending on the level of education, the length of time working on the ranch, the presence of cattle predated in the ranch, and the proportion of the herd affected in attacked ranches (Fig 3). Respondents with higher level of education, particularly those with university studies, showed higher tolerance to the presence of large predators, and perceived less threat to their cattle. Ranchers who had been working on the ranch for over a decade were less willing to receive help for solving cattle predation, and considered that this problem should be address by authorities (Fig 3A). Ranchers who faced cattle predation issues commonly agreed that jaguars posed a threat to their cattle, they expressed concern about the situation on the ranch, and the need for government intervention to address the problem. Among these ranchers, those who experienced a higher proportion of heads predated believed that finding a solution for livestock predation is necessary, and perceived that laws aimed at protecting nature hinder the development of the region (Fig 3B).

## Discussion

Conflicts over wildlife are often very complex and may exhibit different levels of intensity depending on the tolerance towards the species involved. Our results suggest that the conflict between cattle ranching and big cats conservation in the Amazon arc of deforestation could be framed as intermediate, underlying conflict [18], including contradictory and “not in my backyard” attitudes. In other words, ranchers agreed that it was necessary to protect jaguars and pumas, but also showed intolerance to livestock losses in their properties. The conflict in our study area is relevant for the conservation of big cats because they may be killed in response to livestock predation, as occurred in alarming numbers in nearby regions [48]. Indeed, we found that retaliatory killing of jaguars and pumas was the second most frequent response to livestock predation. Killing of carnivores has seriously impacted jaguar populations, translating into one of the primary causes of their decline [25, 26]. Other motivations such as fear, cultural beliefs, or economic interests may significantly contribute to retaliatory killing of carnivores [49, 50].

Ranching practices in our study area did not seem adjusted to the coexistence with large carnivores. The husbandry practices used in the Amazon arc of deforestation, involving large free-ranging herds with calves, were inherently associated with an increased likelihood of predation by jaguars and pumas. These practices allow livestock to enter natural areas, inevitably leading to potential encounters between domestic animals and wildlife, and turning the region into a livestock predation hotspot [22]. In addition, we found that forested areas intermingled in the agricultural matrix were risky regions for livestock, which can be partially explained by the habitat preferences of jaguars and pumas [51, 52]. This was consistent with other studies that have reported proximity to forest as a predictor of livestock predation by jaguars [53]. On the other hand, forested patches are also important for biodiversity conservation [54], thus highlighting a trade-off between conservation and exploitation of natural areas in the region.

Our study reveals the complexities of conservation conflicts, especially when dealing with human attitudes. We found that ranchers did not perceive predation as a significant cause of mortality in their herds. Despite of this, they showed intolerance towards livestock predation, which was occasionally attributed to the breeding of purebred animals. This would ultimately lead ranchers to perceive every loss as significant, even if it is not in numerical terms. In addition, we found that negative attitudes towards big cats were mainly associated with a lower educational background. Our findings are consistent with previous studies elsewhere that identified mortality causes different from predation as the main challenges in extensive cattle ranching [55–57], and highlighted the importance of human factors in shaping attitudes towards carnivores [58–60].

The conflict addressed in this study is predominantly influenced by human factors, rather than solely by livestock predation. Livestock predation would act as a trigger of the conflict, perpetuated by limited knowledge on the importance of large carnivores, the lack of support from the government to mitigate livestock losses, and the perception that conservation laws conditioned the viability of cattle ranching. As a result, focusing solely on reducing attacks will fail to alleviate this conflict, and may even aggravate it [18]. Providing financial compensation could help to alleviate the conflict, especially in such cases where a single loss is highly significant for the ranch. However, compensation schemes need to be applied carefully to avoid an inadequate verification of losses and compensations, which may result in less attention devoted to livestock protection, and even a failure to increase tolerance towards carnivores [8, 61, 62]. Moreover, implementing actions to increase tolerance towards carnivores, such as education on the ecosystem services they provide, could aid in the conservation of big cats in the area. Preserving jaguars and pumas will ensure the maintenance of global biodiversity in tropical forests [63], and together with initiatives to ensure food security and mitigate the adverse effects of agriculture on the environment [64, 65] will encourage the transition towards more sustainable human communities that favour nature conservation.

Our findings include some caveats, related to the nature and age of the dataset. On one hand, data derived from surveys can be noisier and more biased than evidence obtained directly. However, we are confident that our approach provided robust results, due to the high number of ranches included in the survey, together with the ability of ranch managers to identify carnivores’ attacks on livestock through bites or paw prints. Furthermore, data about cattle mortality collected from surveys were compared with data from ranch record books, and statistical tests were applied to identify any bias on the data. On the other hand, several changes have occurred in the study area since the data collection (S6 File). Firstly, some pastures have been replaced by soybean crops, suggesting that cattle ranching may have been reduced, or even ceased, in some properties. Secondly, since 2008, 14% of the remaining forest in the study area has been cleared, mainly for pasture (S6 File), which may have reduced habitat availability for jaguars and pumas, as well as the abundance of wild prey. Consequently, the problem of predation on ranches that currently maintain livestock activity may have been indirectly exacerbated, thus perpetuating the conflict between cattle ranching and carnivore conservation in the study area. The conversion of pastures to croplands in older deforested frontiers [66], such as the study area, is pushing cattle ranching into new intact areas [67], resulting in deforestation there [34, 68] and the consequent appearance of new conflicts. We believe that the drivers of these new conflicts would be very similar to those highlighted in this study, and therefore, our results would allow to develop future mitigation strategies that favour coexistence between cattle ranching and large carnivores along the deforestation frontier.

## Supporting information

S1 File Questionnaire. English translation of Portuguese original questionnaire carried out during 2008 in 129 ranches of the Amazon arc of deforestation.
(DOCX)

S2 File Data. Data used in the manuscript. It contained information collected in questionnaires, as well as the Euclidean distance of the ranch to landscape attributes.
(CSV)

S3 File Ranchers’ profile. Results of the questionnaire about personal information of ranchers: age, education level, length of time living in the region, length of time working on the ranch, and state of origin.
(DOCX)

S4 File Cattle mortality in ranch record books. Comparison between mortality perceived by ranchers and mortality data extracted from record books for two ranches.
(DOCX)

S5 File Statistical modelling. Description of the different models fitted in this study and their results.
(DOCX)

S6 File Recent changes in the study area. Description of land use and land cover changes occurred in the study area between 2008 and 2022.
(DOCX)

S1 Text Request for authorship change(s). (PDF)
