# Supplementary material for: Conflict between cattle ranching and the conservation of jaguar (Panthera onca) and puma (Puma concolor) in the Amazon arc of deforestation
Source: PLoS One. 2024 Nov 20;19(11):e0312077. doi: 10.1371/journal.pone.0312077 (PMC11578515; doi:10.1371/journal.pone.0312077)
Supplement: S1 File — English translation of Portuguese original questionnaire carried out during 2008 in 129 ranches of the Amazon arc of deforestation. (DOCX) [file pone.0312077.s001.docx]

**S1 – Questionnaire**

Questionnaire on livestock predation by jaguars and pumas carried out during 2008 in 129 ranches of the Amazon arc of deforestation (English translation of Portuguese original).

1. **Farm’s characteristics**

1.1 State: 1.2 Municipality: 1.3 Locality:

1.4 UTM coordinates: 1.5 Farm’s name: 1.6 Farm’s age:

1.7 Total area (ha): 1.8 Pasture area (ha):

1.9 Number of workers: 1.10 Number subdivisions:

1. **Interviewee’s profile**

2.1 Age:

( ) 10-16 years old ( ) 17-36 years old ( ) 36-60 years old ( ) older than 61 years

2.2 State of origin:

( ) Tocantins ( ) Pará ( ) Maranhão ( ) Goiás ( ) Bahía ( ) Piauí

( ) Other

2.3 How many years have you lived in the region?

( ) < 5 years ( ) 2-10 years ( ) > 10 years ( ) since I was born

2.4 How many years have you worked at this farm?

( ) < 5 years ( ) 2-10 years ( ) > 10 years ( ) since I was born

2.5 Where do you live?

( ) Farm’s house ( ) Subdivisions’ house ( ) Town

2.6 Studies:

( ) Illiterate ( ) Primary school ( ) Secondary school

( ) University degree ( ) Other

1. **Cattle management**

3.1 Size of cattle herd:

3.2 What phases of cattle breeding are included in this farm?

( ) Breeding ( ) Growing ( ) Fattening

3.3 What type of breeding is used in this farm?

( ) Cattle roam free in the pastures ( ) Cattle are confined in pens

3.4 Has this farm got maternity pastures?

( ) Yes ( ) No

3.5 How many dogs are in the farm?

1. **Cattle loss**

4.1 Which are the main causes of cattle death in this farm?

( ) Drought ( ) Disease ( ) Snake bite ( ) Theft

( ) Predation ( ) Toxic plant ( ) Other

4.2 Have you ever seen jaguar in this farm?

( ) No ( ) Yes, I have seen jaguar ( ) Yes, I have seen jaguar tracks

4.3 Had cattle been predated by felids in you farm?

( ) Yes ( ) No

4.4 How many cattle had been predated during the last year?

4.5 The main species responsible for the attacks is:

( ) Jaguar ( ) Puma ( ) Other

4.6 What kind of cattle are usually predated by jaguar?

( ) Healthy adults ( ) Sick / old adults ( ) Pregnant cows ( ) Young animals

4.7 How do you tell apart predation from other causes of cattle death?

4.8 How many individuals predated by jaguar or puma are you willing to tolerate?

( ) < 5 animals/year ( ) 5-10 animals/year ( ) 10-50 animals/year ( ) >50 animals/year

4.9 Did you have any problems with livestock predation by large cats?

( ) Yes ( ) No

4.10 If yes, in which ones?

( ) Equine ( ) Donkey ( ) Buffalo ( ) Porcine

( ) Ovine ( ) Dogs ( ) Other

4.11 Which method or methods are used to reduce livestock predation in the region?

( ) Cattle management ( ) Poisoning ( ) Cage trap ( ) Poaching

( ) Other

4.12 Which method or methods do you consider best for solving cattle predation?

( ) Payment for the damage caused by predation

( ) Capture and transfer jaguars and pumas to another place

( ) Capture and kill jaguars and pumas, exercising control of their number

( ) Removal of all jaguars and pumas

( ) Other

( ) I do not know

4.13 Have you ever been attacked by a jaguar or a puma or know someone who has?

( ) Yes ( ) No

1. **General attitudes towards big cats**

5.1 Jaguars are a threat to cattle.

( ) Disagree ( ) Do not know ( ) Agree

5.2 Jaguars are a threat to people.

( ) Disagree ( ) Do not know ( ) Agree

5.3 We cannot tolerate livestock predation by jaguars at this ranch.

( ) Disagree ( ) Do not know ( ) Agree

5.4 Livestock predation is an acceptable and natural risk in cattle business.

( ) Disagree ( ) Do not know ( ) Agree

5.5 I would be happier if there were no more jaguars around.

( ) Disagree ( ) Do not know ( ) Agree

5.6 Jaguars need to be protected.

( ) Disagree ( ) Do not know ( ) Agree

5.7 It is necessary to find a solution for livestock predation.

( ) Disagree ( ) Do not know ( ) Agree

5.8 Things are going well in this ranch.

( ) Disagree ( ) Do not know ( ) Agree

5.9 Livestock predation should be addressed by authorities.

( ) Disagree ( ) Do not know ( ) Agree

5.10 Livestock predation should be addressed by each ranch, without external help.

( ) Disagree ( ) Do not know ( ) Agree

5.11 I would like to receive help to solve the problem of predation at this ranch.

( ) Disagree ( ) Do not know ( ) Agree

5.12 Nature/Wildlife in the region is a treasure to be preserved by all people.

( ) Disagree ( ) Do not know ( ) Agree

5.13 I am concerned about nature/wildlife conservation in the region.

( ) Disagree ( ) Do not know ( ) Agree

5.14 Laws that preserve nature hinder the development of the region.

( ) Disagree ( ) Do not know ( ) Agree
