# Supplementary material for: Conflict between cattle ranching and the conservation of jaguar (Panthera onca) and puma (Puma concolor) in the Amazon arc of deforestation
Source: PLoS One. 2024 Nov 20;19(11):e0312077. doi: 10.1371/journal.pone.0312077 (PMC11578515; doi:10.1371/journal.pone.0312077)
Supplement: S3 File — Results of the questionnaire about personal information of ranchers: age, education level, length of time living in the region, length of time working on the ranch, and state of origin. (DOCX) [file pone.0312077.s003.docx]

**S3 - Ranchers’ profile**

129 ranch managers were interviewed on the border between the states of Pará and Tocantins. 55% of the ranchers were middle-aged (36-60 years old), whereas 25 and 20 % were younger or older, respectively (Fig. S3-1A). 55% had primary education. Illiteracy, secondary, or higher education amounted to 11, 25 and 9 %, respectively (Fig. S3-1B). 56 % of ranchers had lived in the region for more than 10 years (Fig. S3-1C), whereas 24 and 20 % had lived in the region between 5-10 years, and less than five years, respectively. Half of ranchers had been working in the ranch where they were interviewed between 2 and 10 years (Fig. S3-1D); 23 and 26 % had been working in the ranch either less than two years, or more than 10, respectively. The surveyed ranchers were born in the Brazilian states of Tocantins (27 %), Goiás (19 %), Maranhão (7.3 %), Pará (4.4 %), Bahia (2.9 %), Piauí (1.5 %), and others (30.7 %).


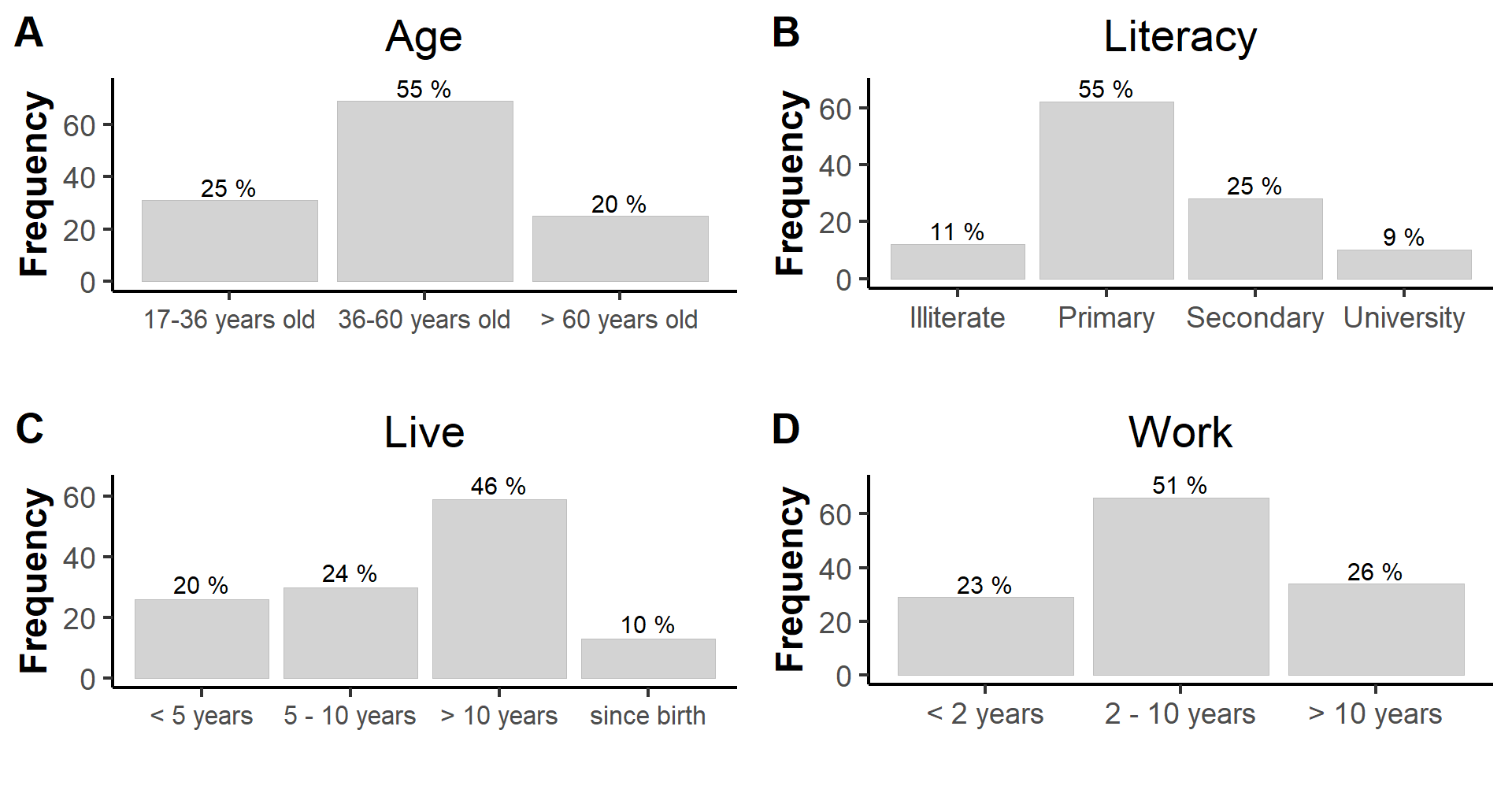
Figure S3-1. Profile of the 129 ranchers interviewed according to age, literacy level, length of time living in the region, and length of time working on the ranch.
