# Supplementary material for: Conflict between cattle ranching and the conservation of jaguar (Panthera onca) and puma (Puma concolor) in the Amazon arc of deforestation
Source: PLoS One. 2024 Nov 20;19(11):e0312077. doi: 10.1371/journal.pone.0312077 (PMC11578515; doi:10.1371/journal.pone.0312077)
Supplement: S4 File — Comparison between mortality perceived by ranchers and mortality data extracted from record books for two ranches. (DOCX) [file pone.0312077.s004.docx]

**S4 - Cattle mortality in ranch record books**

Data from ranch PaB2R010 covered the period January 2002 to December 2007, whereas data from ranch PaB2R016 covered the period January 2001 to December 2007. Ranch managers registered variations in cattle stocks and deaths in record books on a monthly basis. The different causes of cattle mortality were assessed in the field through inspection of carcasses. Six main possible causes of death were identified: disease, ingestion of toxic plants, snake bites, predation by carnivore, accident, and unidentified. Ranch managers or veterinarians looked for signs of predation, like bite marks to the braincase, cervical vertebrae, or throat, scratches and canine punctures on the skin, or signs (paw-prints or scats). This type of analysis did not distinguish between puma and jaguar attacks.

A total of 5,948 cattle carcasses were found and assigned to mortality causes. Snake bites were the most common cause of cattle mortality (Fig. S4-1). The proportion of cattle stock affected by jaguar and puma ranged from 0.13 % to 0.68 % (Fig. S4-2). These results are consistent with the perceived values obtained from the ranchers’ memory of these two ranches, who reported snake bites as the most common cause of cattle mortality and a proportion of herd predated of 0.4 % and 0.7 %.


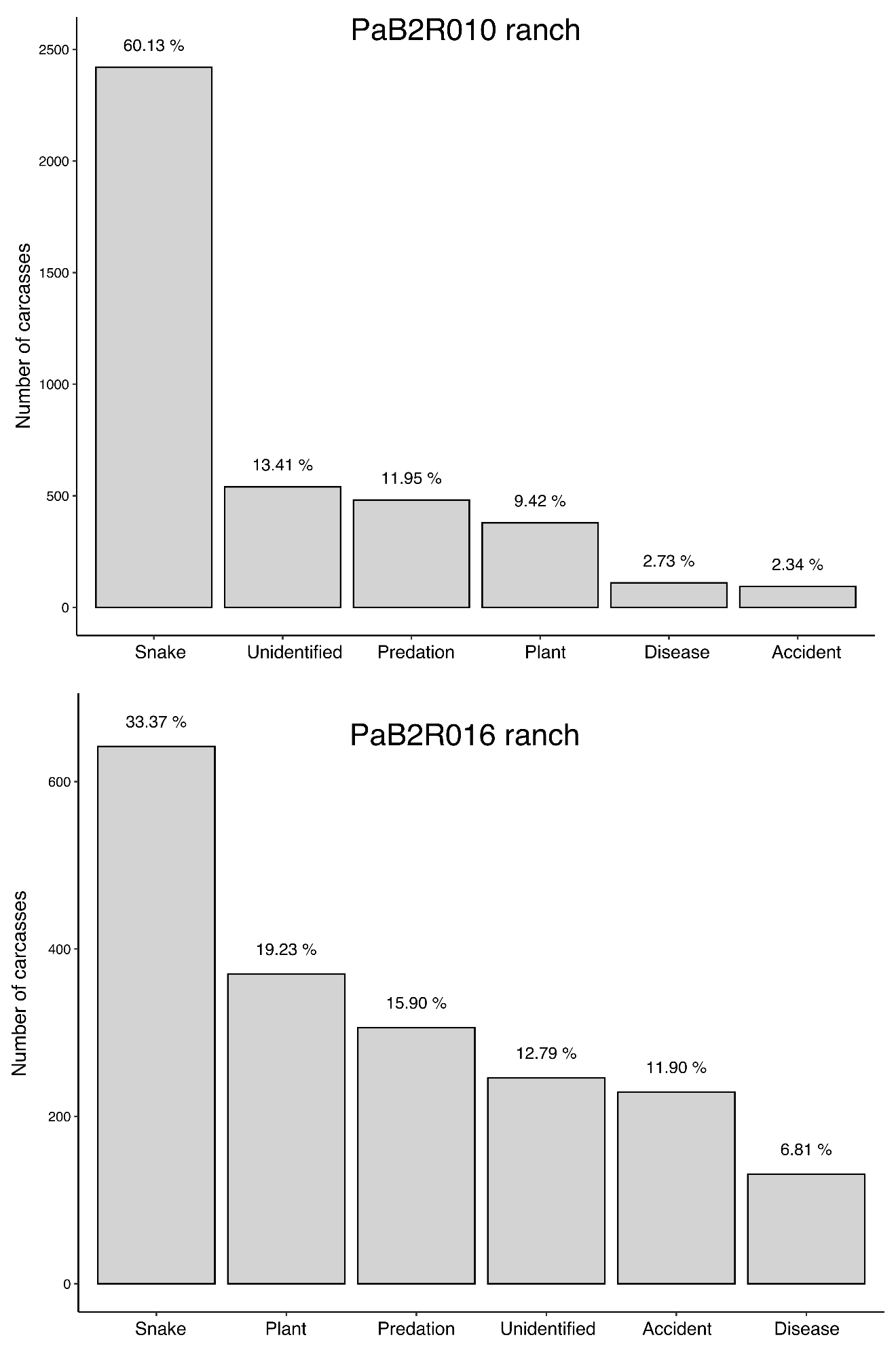


Figure S4-1. Total number of carcasses registered in the logbooks of ranches PAB2R010 and PAB2R016 depending on mortality cause.


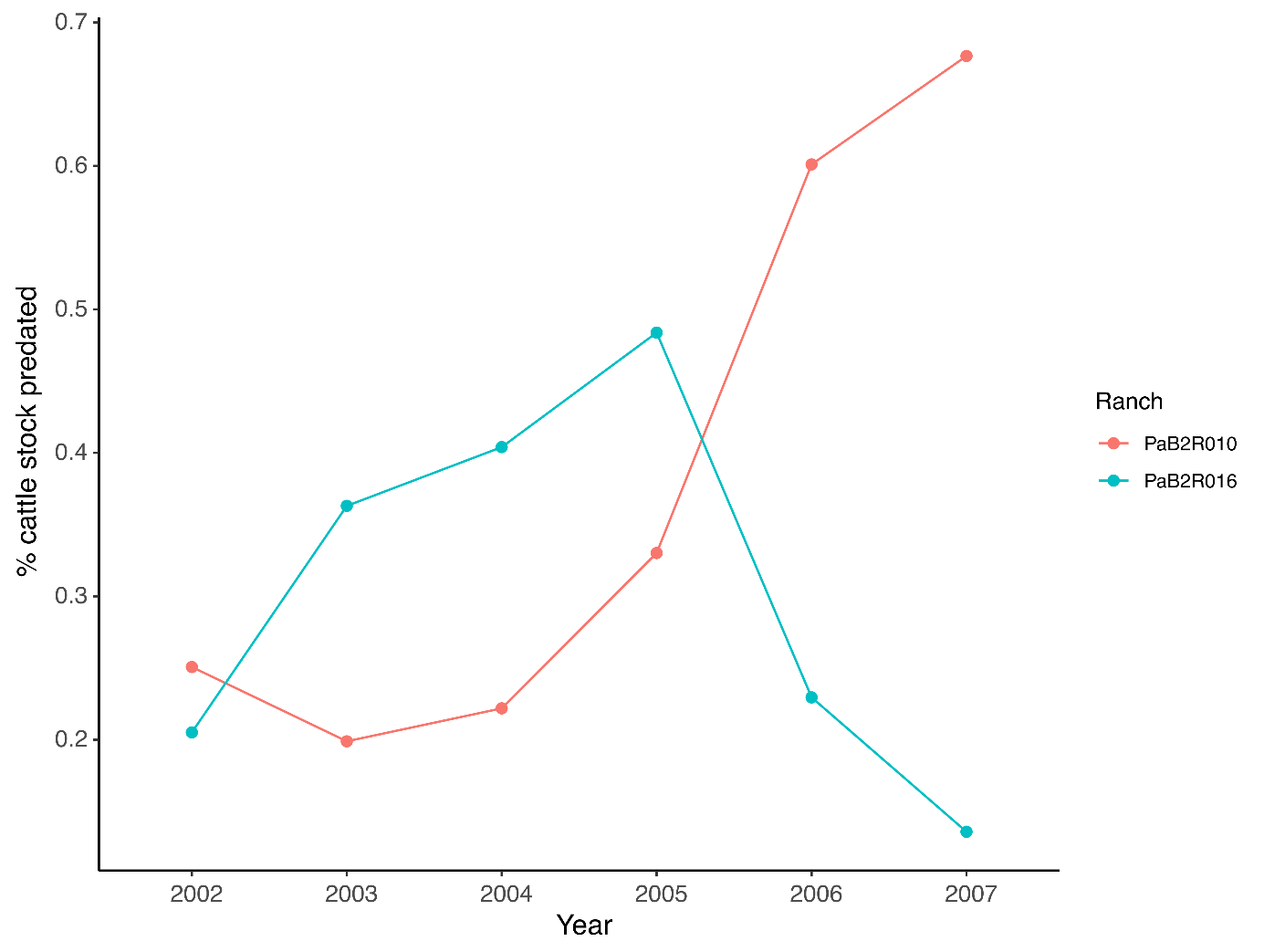


Figure S4-2. Percentage of cattle stock affected by jaguars and pumas’ attacks in ranches PaB2R010 and PaB2R016 during 2002-2007.
