# Supplementary material for: Conflict between cattle ranching and the conservation of jaguar (Panthera onca) and puma (Puma concolor) in the Amazon arc of deforestation
Source: PLoS One. 2024 Nov 20;19(11):e0312077. doi: 10.1371/journal.pone.0312077 (PMC11578515; doi:10.1371/journal.pone.0312077)
Supplement: S5 File — Description of the different models fitted in this study and their results. (DOCX) [file pone.0312077.s005.docx]

**S5 - Statistical modelling**

We fitted four different models:

**Model 1**: binomial GLM on the presence (0/1) of cattle predated by jaguar and puma on ranches including environmental variables as predictors (number of observations = 119)

***Presence cattle predated (0/1)***  *~ Forest + Forest3 + River + National Park + Road + City*

**Model 2:** binomial GLM on the presence (0/1) of cattle predated by jaguar and puma on ranches including cattle management variables as predictors (number of observations = 115).

***Presence cattle predated (0/1)***  *~ Density + Dogs + Workers + Subdivisions + Breeding + Growing + Fattening + Maternity*

**Model 3:** beta-binomial GLM on the proportion of cattle predated in those ranches affected by jaguar and puma including environmental variables as predictors (number of observations = 83). We included the size of the herd (i.e. animals available to be predated by carnivores) as prior weights in the model.

***Proportion cattle predated on ranches with attacks (0/1)***  *~ Forest + Forest3 + River + National Park + Road + City*

**Model 4:** beta-binomial GLM on the proportion of cattle predated in those ranches affected by jaguar and puma including cattle management variables as predictors (number of observations = 80). We included the size of the herd (i.e. animals available to be predated by carnivores) as prior weights in the model.

***Proportion cattle predated on ranches with attacks (0/1)***  *~ Density + Dogs + Workers + Subdivisions + Growing + Fattening + Maternity*

| 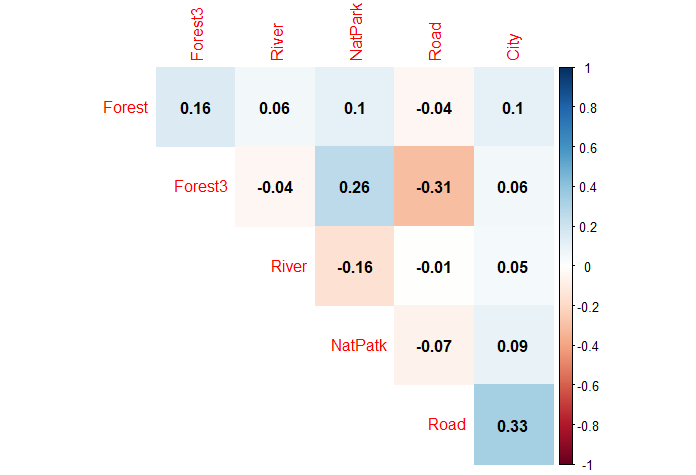  Model 1 | 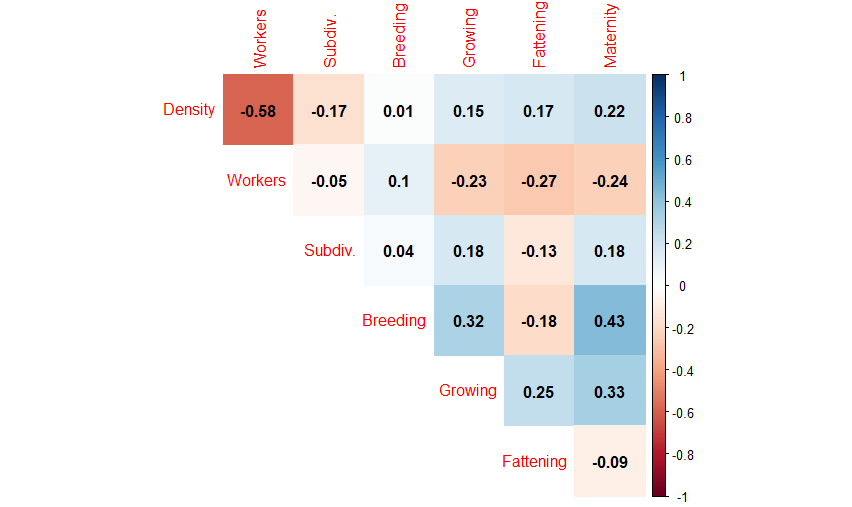  Model 2 |
| --- | --- |
| 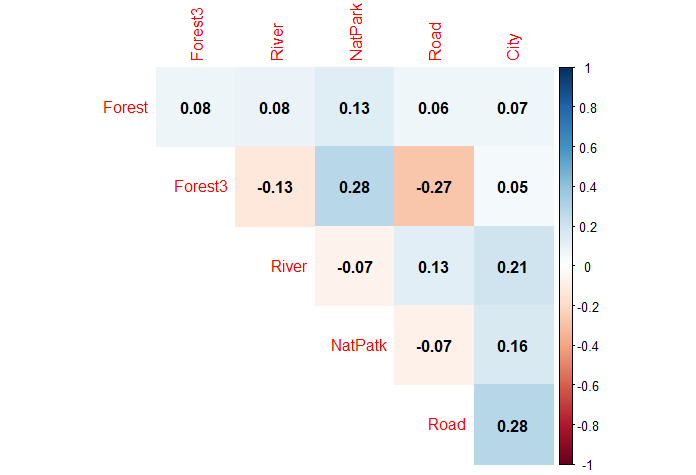  Model 3 | 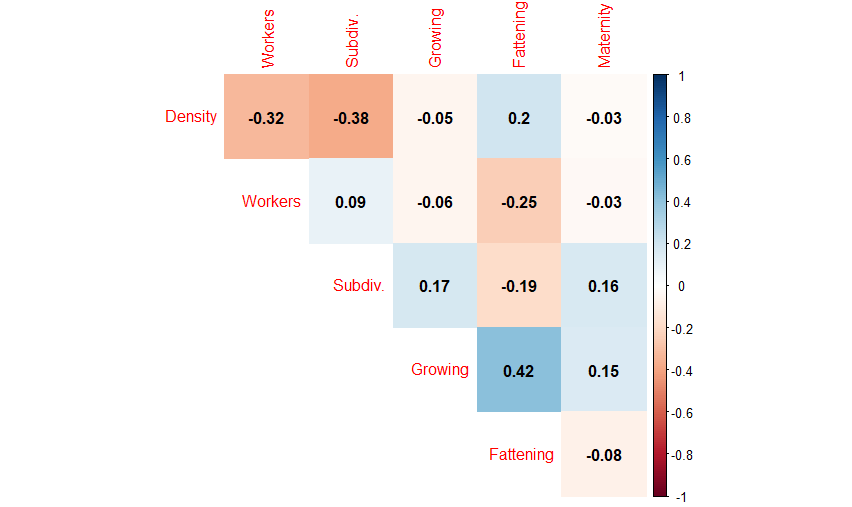  Model 4 |

Fig S5-1. Correlation among the different pairs of predictors for the four models fitted.

Table S5-2. Candidate models fitted on the presence of cattle predated in ranches, and on the proportion of cattle predated in attacked ranches. Models with ΔAIC < 2, ranked by AIC_c_, are shown. For each of the four fitted models, the number of observations (n) was also included.

| **Presence of cattle predated by jaguar and puma in the ranch** | | | | | | |
| --- | --- | --- | --- | --- | --- | --- |
| **Model 1** n = 119 *~ Forest + Forest3 + River + National Park + Road + City* | | | | | | |
| **Candidate models** | | | ***K*^1^** | **AIC_c_^2^** | **ΔAIC_c_^3^** | ***w_i_*^4^** |
| *River + City* | | | 3 | 145.69 | 0 | 0.316 |
| *City* | | | 2 | 146.03 | 0.337 | 0.267 |
| *Forest + River + City* | | | 4 | 146.23 | 0.537 | 0.241 |
| *Forest + City* | | | 3 | 146.85 | 1.161 | 0.177 |
| **Model 2** n = 115 | | *~ Density + Workers + Subdivisions + Breeding + Growing + Fattening + Maternity* | | | | |
| **Candidate models** | | | ***K*^1^** | **AIC_c_^2^** | **ΔAIC_c_^3^** | ***w_i_*^4^** |
| *Density + Subdivisions + Breeding + Fattening + Maternity* | | | 6 | 119.90 | 0 | 0.154 |
| *Density + Subdivisions + Breeding + Fattening* | | | 5 | 119.93 | 0.025 | 0.152 |
| *Density + Subdivisions + Breeding* | | | 4 | 120.29 | 0.381 | 0.127 |
| *Density + Workers + Subdivisions + Breeding* | | | 5 | 120.63 | 0.722 | 0.107 |
| *Density + Subdivisions + Breeding + Maternity* | | | 5 | 120.63 | 0.728 | 0.107 |
| *Density + Workers + Subdivisions + Breeding + Fattening* | | | 6 | 121.11 | 1.202 | 0.085 |
| *Density + Subdivisions + Breeding + Growing + Fattening + Maternity* | | | 7 | 121.41 | 1.503 | 0.073 |
| *Density + Workers + Subdivisions + Breeding + Maternity* | | | 6 | 121.52 | 1.618 | 0.069 |
| *Density + Workers + Subdivisions + Breeding + Fattening + Maternity* | | | 7 | 121.62 | 1.711 | 0.066 |
| *Density + Subdivisions + Breeding + Growing + Fattening* | | | 6 | 121.80 | 1.891 | 0.060 |
| **Proportion of cattle predated in ranches attacked by jaguar and puma** | | | | | | |
| **Model 3** n = 83 *~ Forest + Forest3 + River + National Park + Road + City* | | | | | | |
| **Candidate models** | | | ***K*^1^** | **AIC_c_^2^** | **ΔAIC_c_^3^** | ***w_i_*^4^** |
| *Forest + National Park* | | | 4 | 671.95 | 0 | 0.338 |
| *Forest + Forest3 + National Park* | | | 5 | 672.26 | 0.31 | 0.291 |
| *Forest + River + National Park* | | | 5 | 672.99 | 1.04 | 0.202 |
| *Forest + Forest3 + River + National Park* | | | 6 | 673.38 | 1.42 | 0.167 |
| **Model 4** n = 80 | *~ Density + Workers + Subdivisions + Growing + Fattening + Maternity* | | | | | |
| **Candidate models** | | | ***K*^1^** | **AIC_c_^2^** | **ΔAIC_c_^3^** | ***w_i_*^4^** |
| *Density + Workers + Growing* | | | 5 | 635.31 | 0 | 0.084 |
| *Workers + Fattening* | | | 4 | 635.52 | 0.22 | 0.076 |
| *Workers + Fattening + Maternity* | | | 5 | 635.81 | 0.50 | 0.066 |
| *Workers + Growing + Fattening* | | | 5 | 636.15 | 0.84 | 0.056 |
| *Density + Fattening* | | | 4 | 636.16 | 0.85 | 0.055 |
| *Density + Workers + Growing + Fattening* | | | 6 | 636.19 | 0.88 | 0.054 |
| *Density + Workers + Fattening + Maternity* | | | 6 | 636.27 | 0.96 | 0.052 |
| *Density + Workers + Fattening* | | | 5 | 636.27 | 0.96 | 0.052 |
| *Density + Growing + Fattening* | | | 5 | 636.39 | 1.08 | 0.049 |
| *Workers + Growing* | | | 4 | 636.43 | 1.12 | 0.048 |
| *Workers + Subdivisions + Growing* | | | 5 | 636.50 | 1.19 | 0.046 |
| *Density + Fattening + Maternity* | | | 5 | 636.50 | 1.20 | 0.046 |
| *Density + Growing* | | | 4 | 636.71 | 1.41 | 0.042 |
| *Density + Workers + Subdivisions +Growing* | | | 6 | 636.83 | 1.52 | 0.039 |
| *Fattening* | | | 3 | 636.95 | 1.64 | 0.037 |
| *Density + Workers + Growing + Maternity* | | | 6 | 637.06 | 1.75 | 0.035 |
| *Workers + Subdivisions + Growing + Fattening* | | | 6 | 637.19 | 1.88 | 0.033 |
| *Workers + Subdivisions + Fattening* | | | 5 | 637.20 | 1.89 | 0.033 |
| *Workers + Subdivisions + Fattening + Maternity* | | | 6 | 637.24 | 1.93 | 0.032 |
| *Workers + Growing + Fattening + Maternity* | | | 6 | 637.26 | 1.95 | 0.032 |
| *Density + Workers + Growing + Fattening + Maternity* | | | 7 | 637.26 | 1.95 | 0.032 |

^1^K, number of parameters in the model (includes intercept term)

^2^AIC_c_, AIC estimate corrected for small sample size

^3^ΔAIC_c_, difference of the AIC_c_ values between the most supported model and the given model

^4^*w_i_*, Akaike weight for each model
