# Supplementary material for: Conflict between cattle ranching and the conservation of jaguar (Panthera onca) and puma (Puma concolor) in the Amazon arc of deforestation
Source: PLoS One. 2024 Nov 20;19(11):e0312077. doi: 10.1371/journal.pone.0312077 (PMC11578515; doi:10.1371/journal.pone.0312077)
Supplement: S6 File — Description of land use and land cover changes occurred in the study area between 2008 and 2022. (DOCX) [file pone.0312077.s006.docx]

**S6 - Recent changes in the study area**

We approximated the extent of the study area by creating buffers around the ranches based on their size and drawing a convex polygon enclosing these boundaries (Fig. S6-1). In order to gain insight into the changes that have occurred within the study area, data pertaining to land use and land cover were obtained for the years 2008 (when the study was conducted) and 2022 (the present time). This information was obtained from Map Biomas Project - Collection 8 of the Annual Land Use Land Cover Maps of Brazil [access on 9^th^ September 2023] <https://brasil.mapbiomas.org/>. The rasters obtained for the study area were subsequently transformed into shapefile, thus facilitating the calculation process.

We obtained the extension of each type of land use/land cover for 2008 and 2022 (Table S6-1). Between 2008 and 2022, forests and pastures lost 2.8 % and 2.4 % of their area, respectively. The land use that increased the most during this period was soybean crops, with a 22-fold increase in area (from 0.4 to 8.8 %, Table S6-1).

To detect deforestation during 2008-2022 period, we selected areas that appear classified in Map Biomas as “3” = “Forest Formation” in 2008 and that were no longer identified as such in the 2022 coverage. During 2008-2022 period, 14 % of the 2008 forest cover (894.545 km^2^) was deforested and converted to other land uses, mainly pastures (Fig. S6-2).


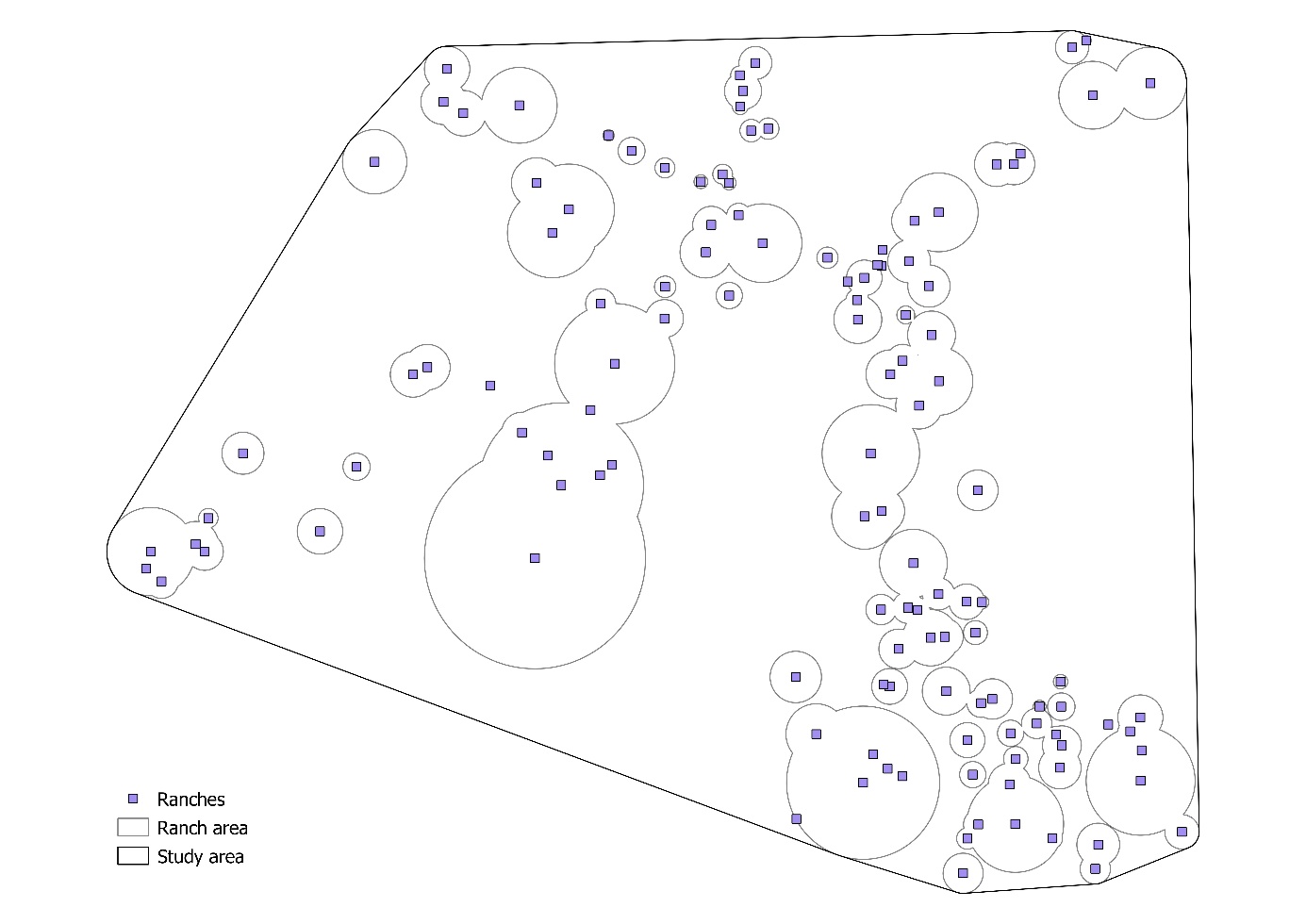


Figure S6-1. Study area of 19,211 km2 delimited by ranches. All ranches visited and their respective area are also showed.

Table S6-1. Area (km^2^, %) of each land cover and land use in 2008 and 2022.

|  |  | **2008** | | **2022** | |
| --- | --- | --- | --- | --- | --- |
| **ID** | **Land use / Land cover** | **Area (km^2^)** | **%** | **Area (km^2^)** | **%** |
|  | Forest |  |  |  |  |
| 3 | Forest Formation | 6,297 | 32.8 | 5,755 | 30 |
| 4 | Savanna Formation | 3,307 | 17.2 | 2,482 | 12.9 |
| 6 | Floodable Forest | 7 | 0.0 | 4 | 0.0 |
|  | Non Forest Natural Formation |  |  |  |  |
| 11 | Wetland | 1,147 | 6.0 | 1,062 | 5.5 |
| 12 | Grassland Formation | 287 | 1.5 | 249 | 1.3 |
| 29 | Rocky Outcrop | 23 | 0.1 | 23 | 0.1 |
|  | Farming |  |  |  |  |
| 15 | Pasture | 7,455 | 38.8 | 6,988 | 36.4 |
| 39 | Soybean | 79 | 0.4 | 1,697 | 8.8 |
| 41 | Other Temporary Crops | 40 | 0.2 | 98 | 0.5 |
| 9 | Forest Plantation | 4 | 0.0 | 55 | 0.3 |
| 21 | Mosaic of uses | 138 | 0.7 | 341 | 1.8 |
|  | Non Vegetated Area |  |  |  |  |
| 24 | Urban Area | 17 | 0.1 | 18 | 0.1 |
| 30 | Mining | 0 | 0.0 | 8 | 0.0 |
| 25 | Other Non-Vegetated Areas | 80 | 0.4 | 181 | 0.9 |
|  | Water |  |  |  |  |
| 33 | River, Lake and Ocean | 329 | 1.7 | 251 | 1.3 |

*
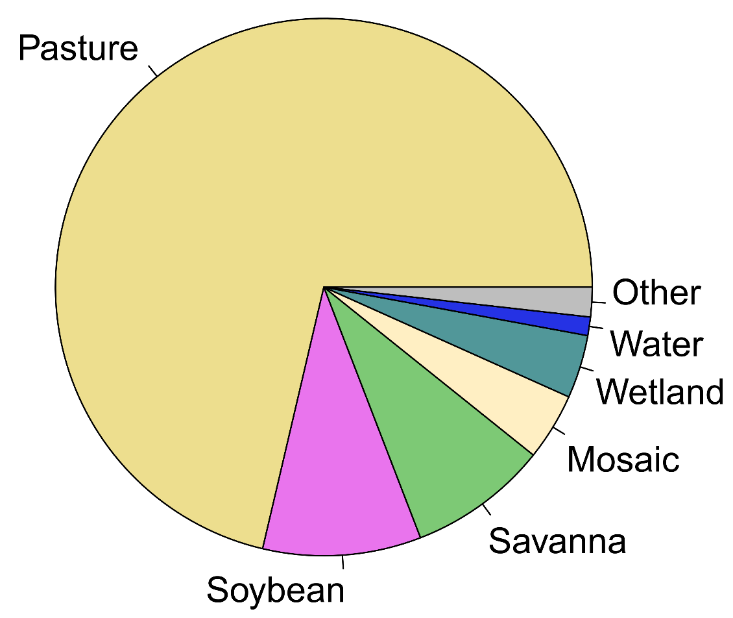
*

Figure S6-2. Land uses of deforested areas between 2008 and 2022.
